# Knee Joint Tissues Effectively Separate Mixed Sized Molecules Delivered in a Single Bolus to the Heart

**DOI:** 10.1038/s41598-018-28228-w

**Published:** 2018-07-06

**Authors:** Lucy Ngo, Lillian E. Knothe, Melissa L. Knothe Tate

**Affiliations:** 1 MichBio Team, Graduate School of Biomedical Engineering, University of New South Wales, Sydney, Australia; 2 Paul Trainor Chair of Biomedical Engineering, Graduate School of Biomedical Engineering, University of New South Wales, Sydney, Australia

## Abstract

The role of molecular size selectivity in the onset and progression of osteoarthritis (OA), a degenerative disease of the musculoskeletal system and the most common cause of disability in aging adults, is unknown. Here we delivered a mixture of Texas-red (70 kDa), and Rhodamine-green (10 kDa) tagged, dextrans of neutral charge in a single bolus via heart injection to middle aged (8–10 months) and aged (17–19 months) Dunkin-Hartley Guinea pigs, a natural model for OA. We quantified tracer transport in serial-sectioned, cryofixed block specimens after five minutes’ circulation. A remarkable separation of the molecules was observed in serial fluorescent images of whole joint sections. The larger, 70 kDa red tracer was abundant in the marrow cavity albeit less prevalent or absent in the bone, cartilage, meniscus and other tissues of the joint. Tissues of the meniscus, ligament, and tendon exhibited abundant 10 kDa tracer; volumes of tissue containing this molecular tracer were significantly lower in older than in younger animals. Surprisingly, muscle fiber bundles exhibited little fluorescence, while their bounding fasciae fluoresced either red or green. Small caliber channels through the articular cartilage appeared to show a degree of green fluorescence not observed in the surrounding cartilage matrix. This study opens up new avenues for study of musculoskeletal physiology in health and disease as well as new strategies for drug delivery.

## Introduction

Tissues of the brain^1^, lung^2^ and gut^3^ exhibit the functional barrier property of molecular size selectivity, which is essential for normal physiologic function throughout life^4^. A recent study correlates aging with increased intestinal permeability to solutes but not macromolecules, which modulates “innate mucosal immune responsiveness [of] elderly humans“^3^. Osteoarthritis (OA), a degenerative disease of the musculoskeletal system and the most common cause of disability in aging adults, is a multifactorial disease involving complex interactions between tissues of articular joints and the immune system. The role of size selectivity in the onset and progression of OA is unknown. Molecular transport between tissue compartments of the musculoskeletal system occurs via blood, interstitial fluid and lymphatic flow^5,6^. An understanding of changes to molecular transport associated with aging and disease of musculoskeletal organs, such as the knee joint, may have important implications for unraveling the etiology and pathogenesis of osteoarthritis (OA) and other degenerative diseases associated with aging.

The resident cells of different musculoskeletal tissues making up a given articular joint (*i.e*. cartilage, subchondral bone, bone, meniscus, synovium) depend on molecular transport via blood flow, as well as interstitial transport between vascular and avascular tissues, to survive. The closed circulatory system of vertebrates provides a means for molecular transport to the respective vascularized tissues of the musculoskeletal system via the blood, with delivery to and from the heart via respective venous and arterial systems and interstitial fluid shunting via the lymphatic system^7^. Yet it is currently not known how molecules such as pro-inflammatory cytokines, growth factors, nutrients and proteolytic enzymes are transported from the blood, to and between different tissue compartments within articular joints.

It is currently unknown whether the bounding layers of bone and cartilage (periosteum, subchondral bone) actively modulate transport into and out of tissue compartments of the joint and musculoskeletal system. The expression of ZO-1 proteins by periosteal cells^8^ may implicate tight junctions as a means to control permeability across the periosteum, the outer bounding envelope of all bone surfaces not covered in cartilage. Furthermore, a recent study implicates the relative balance of two tight cell-cell adhesion architectures (‘zippers’ that seal surfaces and ‘buttons’ that enable controlled infiltration of lymph under controlled flow or pressure gradients) as a putative means to control relative drainage of interstitial fluid via the lymphatic system, in particular during development, and in (patho-)physiology^9^. The relative contributions of arterial and venous circulation, as well as the lymphatic circulation, to interstitial flow and molecular transport in the tissues of the musculoskeletal system are not clearly delineated.

Furthermore, diffusion and convection are putative transport mechanisms to and from avascular articular joint cartilage tissue^10–12^, although small channels have been postulated to physically link cartilage to the subchondral bone interface^13^. Assessment of the transport to/from and between tissue compartments via blood and interstitial fluid flow necessitates the use of an imaging modality that allows for seamless imaging of transport across organ, tissue, cellular and molecular length scales. Visualization of molecular transport at organ, tissue, cellular, and molecular length scales in healthy and OA tissue compartments is expected to uncover the underpinnings of OA onset and progression. Hence, we aimed to quantify differences in transport between tissue compartments of the knee joint, to test the hypothesis that boundary tissues exhibit molecular size dependent barrier properties similar to those observed in the brain, lung and gut.

## Results and Discussion

We assessed molecular communication in knee joints comprising bone, cartilage, and the vascular system, with particular interest in transport across tissue boundaries including the bone-cartilage interface of the joint. We injected, in a single bolus to the heart of anaesthetized Guinea pigs, a mixture of fluorescent-red tagged, 70 kDa, and fluorescent-green tagged, 10 kDa, dextrans of neutral charge and allowed for five minutes circulation before sacrifice. Molecular tracer sizes were chosen based on previous experiments showing molecular sieving in unloaded and loaded cortical bone tissue^14–17^, ligament^18^, and cartilage^13^ and in consideration of physiologically relevant biological analogs in a similar range of molecular sizes. The Dunkin-Hartley strain of Guinea pig serves as a model for naturally occurring osteoarthritis, where cartilage lesions increase in frequency and size with increasing age^19–21^. Two cohorts were studied, including skeletally mature male GP at 8–10 (corresponding to middle age in humans) and 17–19 (corresponding to aging humans) months^20^. After five minutes’ circulation, animals were sacrificed and knee joints were immediately frozen *in vivo* in OCT medium using liquid nitrogen, after which cryosectioning and high resolution, three-dimensional imaging of block faces was carried out in reflection and fluorescence modes.

**Figure 1:**
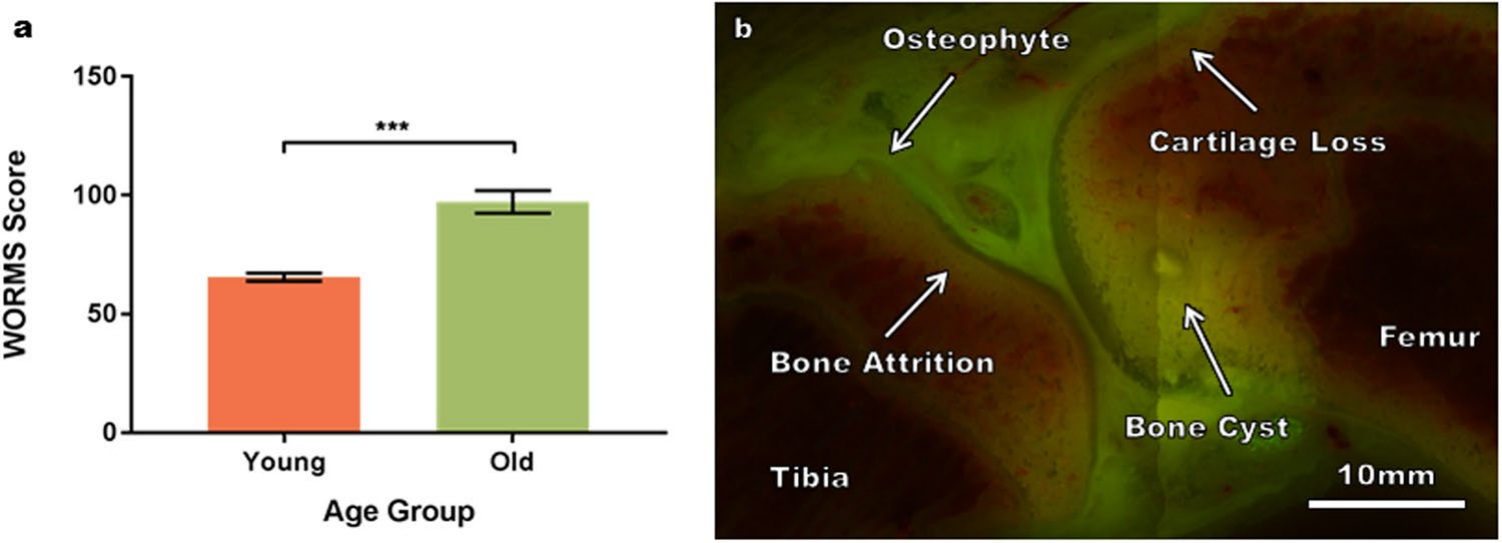
WORMS assessment method and characteristic OA features. (**a**) Semi-quantitative multi-feature OA assessment demonstrates significantly greater custom summed WORMS scores in older animals than in younger animals (+/− standard error bars, p = 0.0006). Statistical significance was analyzed by unpaired t test with Welch’s correction using Graph-Pad Prism 6 (GraphPad Software, Inc.). (**b**) Representative OA features used in the WORMS analysis including osteophytes, cartilage loss, bone cysts and bone attrition in a single serial sectioned slice.

Specimen images were assessed analogous to the custom summed whole-organ magnetic resonance imaging scores (WORMS)^22^, a semi-quantitative multi-feature evaluation used to assess disease progression in human patients. This comprised of individual scores for five tibiofemoral sub-regions (central and posterior femoral and anterior, central and posterior tibial) for each of the medial and lateral compartments for knee OA features. The features assessed in each sub-region, included cartilage morphology (scored out of six), bone cysts and bone attrition (scored out of three) and osteophytes (scored out of seven), for a maximum total score of 190. The total WORMS score for younger animals was significantly lower than that of the older age group (Fig. 1a). All animals displayed each OA feature in at least one sub-region (Fig. 1b). The distribution of features showed no partiality towards either the medial or lateral compartment and there was little indication that the scores of individual features were significantly different between groups with the exception of bone attrition. This is consistent with the multifactorial nature and heterogeneity of OA.

In addition, a coronal view of the imaging data set was constructed, analogous to anterior-posterior radiographs, for qualitative analysis with the commonly used Kellgren-Lawrence (KL) system for classification of knee OA^23^. The characteristics of interest were osteophytes, bone end deformity and cartilage loss, representative of joint space narrowing. The younger animals exhibited multiple osteophytes and varied regions of both partial and full depth cartilage loss with some deformity of tibial plateau; the mean KL score was 2.67 for the younger animals. The older animals also exhibited multiple osteophytes but areas of cartilage loss were larger with marked bone-on-bone cartilage erosion; the mean KL score was four for the older animals.

**Figure 2:**
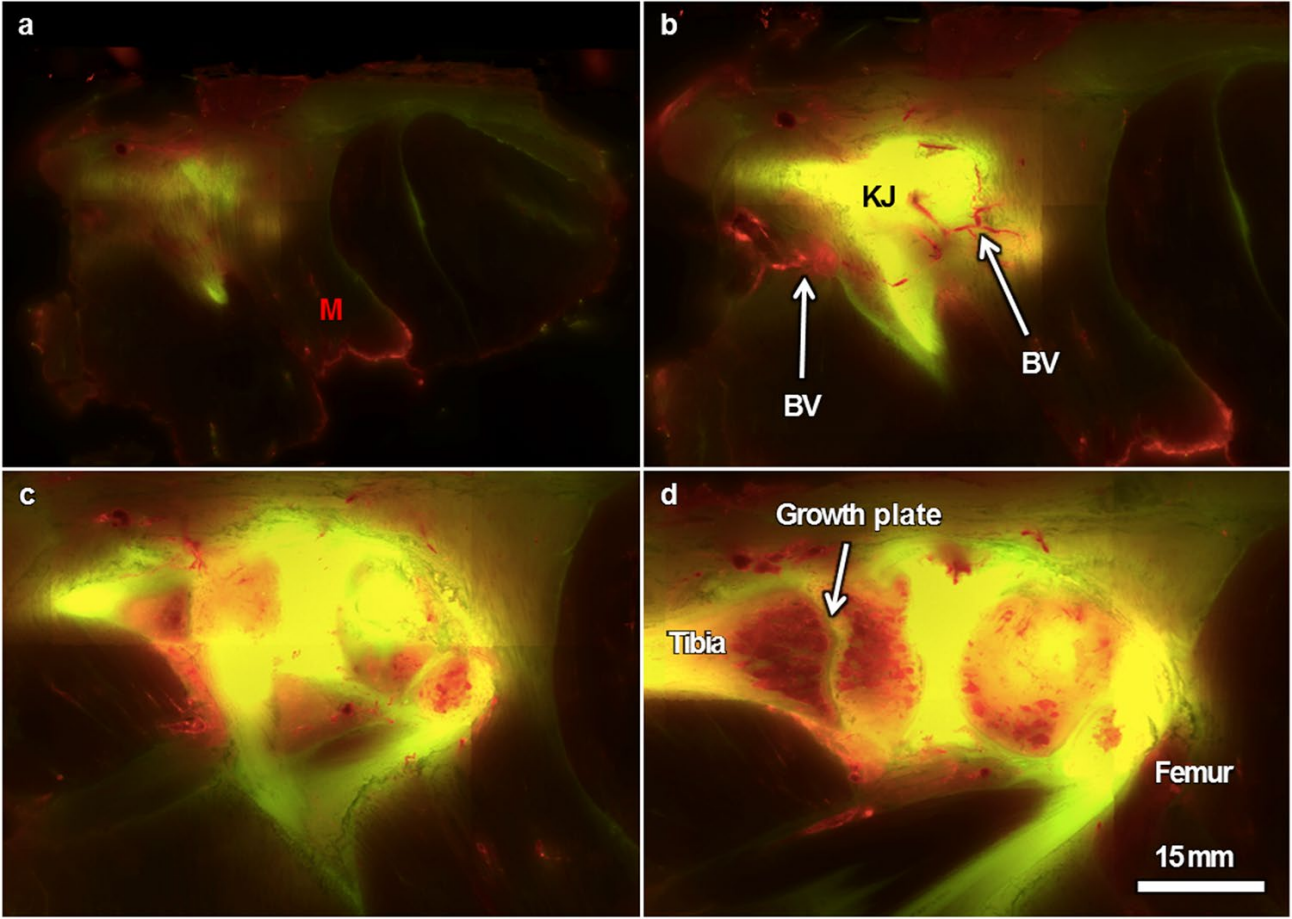
Representative serial images through the knee joint (KJ) and surrounding musculature (M) of a knee from the younger cohort. Sections are 40 microns apart in the cutting plane. (**a**) Just above the joint capsule (green, visible through the muscle), muscle tissue exhibits little fluorescence; muscle fasciae exhibit the 70 kDa red tracer. (**b**) Sectioning further into the block, blood vessels (BV, arrows) exhibit the large molecular weight red tracer and the bone, joint capsule as well as ligaments exhibit the 10 kDa green tracer. (**c**) Cutting further into the block, the 70 kDa red tracer is still visible in blood vessels as well as in marrow cavities of the trabecular bone. (**d**) The anterior meniscus also exhibits the larger molecular weight red tracer in vascularized areas (in contrast to the avascular human meniscus, the anterior Guinea pig meniscus is vascularized) but not in the avascular posterior meniscus, which is overexposed in this image (exposure time 2000 ms).

**Figure 3:**
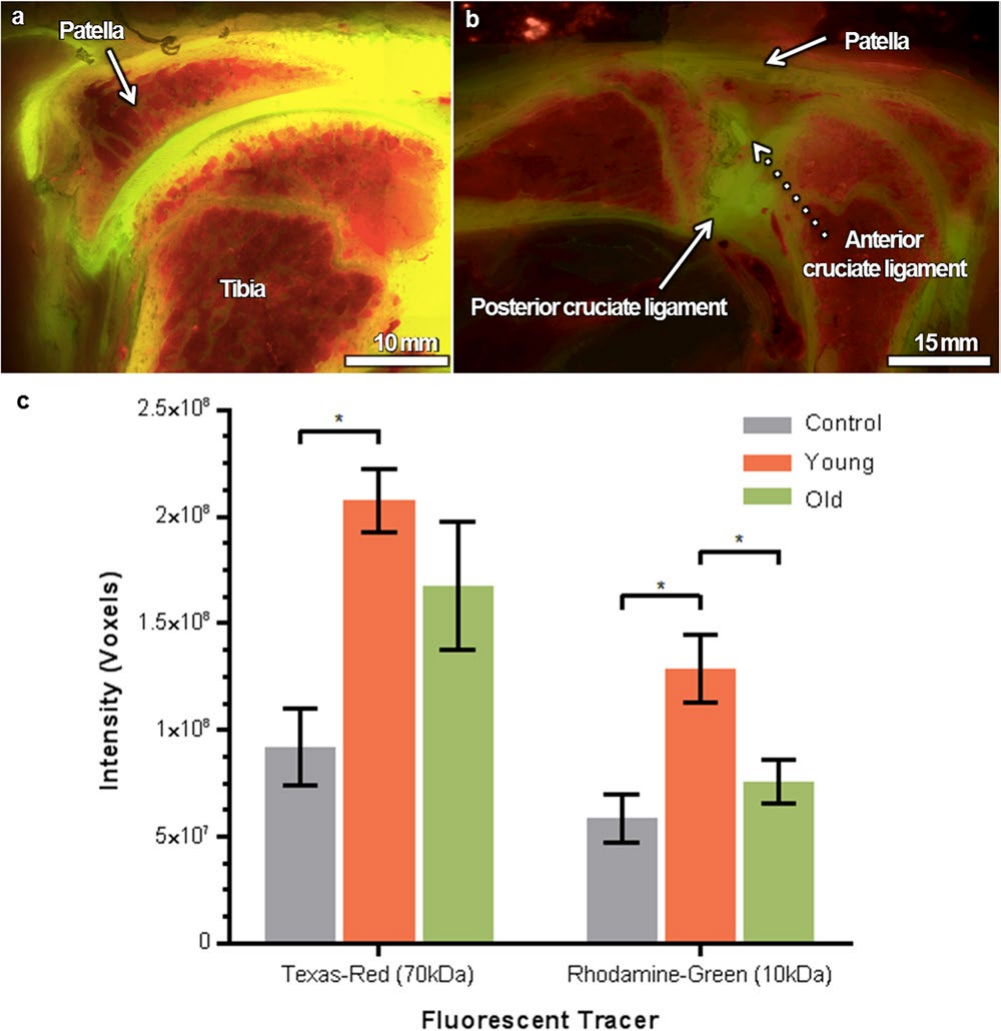
Quantification of tracer distribution. (**a**) Episcopic block face images (left), fluorescent images (middle), and resulting 3D reconstructions of the knee joint (right) are segmented to quantify tracer distributions. (**b**) Pixel (and voxel, Fig. 3g) quantification of red and green tracers demonstrate that older animals exhibit low tracer concentrations similar to those of baseline control animals while younger animals exhibit significantly higher concentrations. (**c**) Contrasting concentrations of tracers observed in different articular compartments. Younger animals exhibited significantly greater red tracer concentrations that the control animals (p = 0.0184) and green tracer in the control and older cohort (p = 0.0143 and p = 0.0201, respectively). Statistical significance was analyzed by unpaired T-test with Welch’s correction using Graph-Pad Prism 6 (GraphPad Software, Inc.).

**Figure 4:**
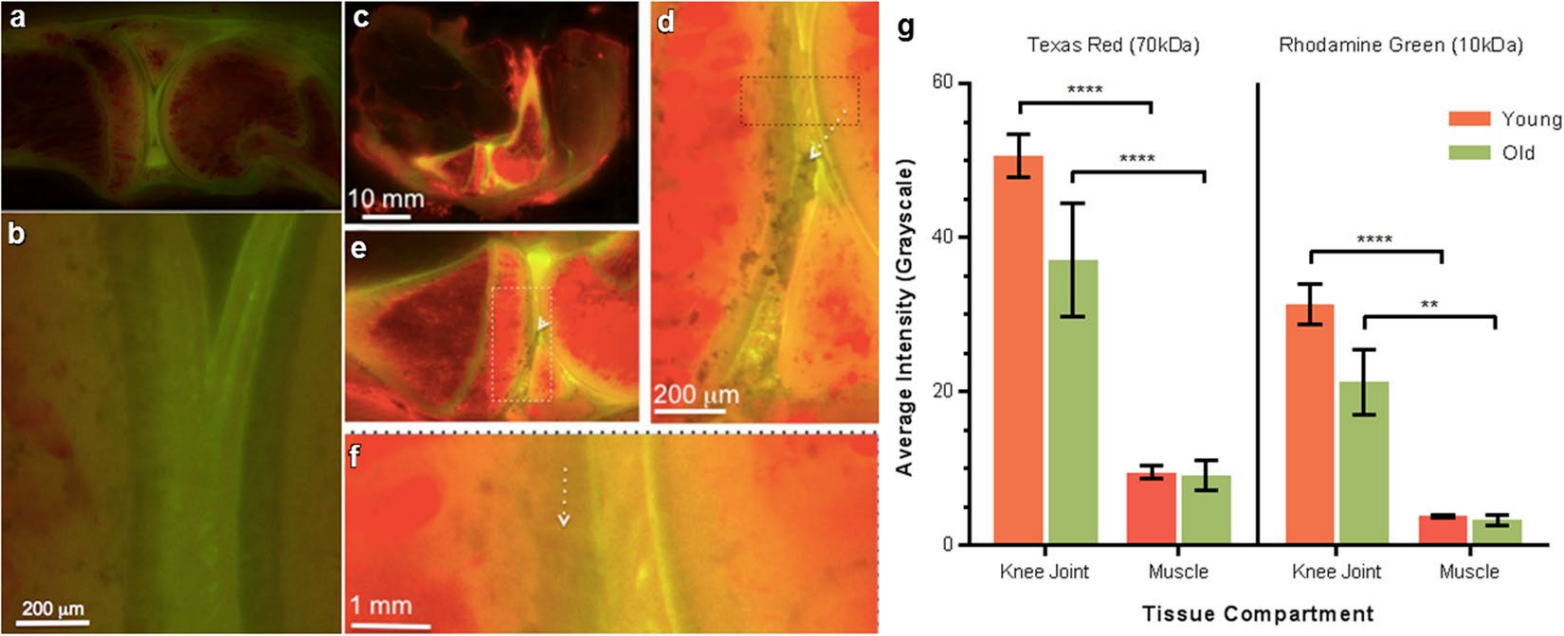
Comparison of transport in representative knee joints of younger (**a**,**b**) and older (**c**–**f**) cohorts. Younger cohort imaged with 500 ms exposure time. Older cohort imaged with 2000 ms exposure time. (**a**) In the younger cohort, partitioning of tracers is clear, where the 70 kDa red tracer is visible in the marrow cavities and vascularized areas of subchondral bone and anterior meniscus. The 10 kDa green tracer is abundant in the joint capsule and nonvascularized posterior meniscus. (**b**) In the younger cohort, the articular cartilage is grey, appearing to exclude both tracers except across small channels bridging across the cartilage from the joint capsule to the subchondral bone. (**c**,**d**) In contrast, in the older cohort the tissue compartments of the knee exhibit similar molecular size partitioning with decreased tracer penetration compared to younger animals. Further, the articular cartilage exhibits lesions typical for osteoarthritis, a condition that develops spontaneously in Dunkin-Hartley guinea pigs. (**e**) Lesion areas exhibit disrupted transport in general and less apparent connectivity across the articular cartilage, between the joint capsule and the subchondral bone. (**f**) Digitally zoomed in view suggests small caliber channels across the width of cartilage. (**g**) Voxel quantification of red and green tracers demonstrate significant differences in tracer concentrations of both the 10,000 Da green and 70,000 Da green tracers attributable to tissue type as well as age of the animal. In general, muscle exhibited significantly less red and green tracer than did tissues of the knee joint for both younger (+/− standard error bars, p < 0.0001 and p < 0.0001, respectively) and older groups (+/− standard error bars, p < 0.0001 and p = 0.0063, respectively). In addition, older animals exhibit low tracer concentrations similar to those of baseline control animals while younger animals exhibit significantly higher concentrations. Statistical significance was analyzed by three-way ANOVA using Graph-Pad Prism 6 (GraphPad Software, Inc.).

Respective tissues of the knee joint exhibited size selective partitioning of molecules, even though the molecular tracers were injected to the heart as a single, mixed bolus of sizes. Although dextrans were delivered as a single mixture of green small and red large molecular weights, a remarkable separation of the two sizes of molecules (red and green) was observed in fluorescent images of whole joint sections, indicative of size separation by the tissues of the joint and greater musculoskeletal system (Figs 2–4, Supplementary Animation 1–3). Volume measures (voxels) of red and green tracers demonstrated that older animals exhibit fluorescent tracer concentrations lower than those of the younger cohort. Younger animals exhibited significantly higher concentrations of the green 10 kDa tracer (Fig. 4g). Remarkably, muscle tissues exhibited little fluorescence, with bounding fasciae lighting up either red or green (Figs 2, 3b, 4c and Supplementary Animation 1, 2). Articular cartilage showed little evidence of either tracer (Fig. 4b), while tissues of the meniscus, ligament, and tendon lit up with the 10 kDa (green) tracer (Figs 3, 4). The 10 and 70 kDa tracers were colocalized (yellow) in bone, with a surprising exclusion of the higher molecular weight tracer in bounding tissues, including the periosteum, growth plate, and cartilage. The 70 kDa tracer (red) was abundant in the marrow space. Small caliber channels through the articular cartilage, showing higher green intensity than surrounding cartilage matrix, were readily observable in knees of younger animals but were barely discernable in knees of the older cohort (Figs 3, 4f).

These data indicate that tissues of the musculoskeletal system exhibit active compartmentalization similar to other organ systems of the body. Our results point to a previously unappreciated capacity of different musculoskeletal compartments to separate molecules based on size. These data further implicate barrier membrane functions of the periosteum, growth plate and synovial membrane. Interestingly, recent studies demonstrate higher permeability of the periosteum in the bone to muscle direction than *vice versa*^24^. Furthermore, after five minutes circulation in anaesthetized animals, molecular transport to musculature surrounding the knee joint was markedly less than that observed in the tissue compartments of the bone and joint, providing further evidence for the suggestion that bone provides an important role in maintenance of muscle^25,26^. Finally, articular lesions exhibit disruption to molecular transport, which may have important implications in the etiology and pathogenesis of osteoarthritis, the most common musculoskeletal disease and disability in aging adults. This is in stark contrast to studies reporting increased transport in OA joints for very small molecular weight tracers (less than 1 kDa)^13,25^. Additionally, recent literature implicates the role of intraarticular structures on the pathogenesis of OA, *e.g*. inflammation of the synovium^27,28^.

Our current working hypothesis is that inflammatory cytokines actively modulate molecular barrier properties of boundary tissues, enabling active control of transport between tissue compartments, similar to that observed in the brain, lung and gut^29–31^. Furthermore, extracellular matrices of different joint tissues exhibit independent filttration properties; we hypothesize that the relative compositions of ECMs in health and disease can effectively shift the balance between transport of matrix degrading proteolytic enzymes (of molecular weight less than 50 kDa) and their inhibitors that are transported via the blood and are too large to penetrate healthy tissues^5^. It is currently not known how molecules such as inflammatory cytokines, growth factors, nutrients and proteolytic enzymes are transported from the blood, to and between tissue compartments within articular joints. Assessment of the transport to and from and betweeen tissue compartments via blood and interstitial fluid flow necessitates the use of an imaging modality such as that presented here, which allows for seamless imaging of transport across organ, tissue, cellular and molecular length scales. Given previous studies that demonstrate size and charge separation in the different caliber porosities of bone^16,17^, as well as studies showing active barrier function of boundary tissues (*e.g*. periosteum), these studies may open a new avenues to elucidate joint physiology in health and disease, the role of intraarticular and extraarticular transport of inflammatory cytokines on tissue permeability, as well as physical (exercise) and chemical (pharmaceutical) measures to modulate joint tissue health with age.

## Materials and Methods

### Intravital tracer injection

This study was approved by the Case Western Reserve University IACUC (Protocol #2012-0100). All experiments and methods were performed in accordance with relevant guidelines and regulations of this IACUC. Three anaesthetized 8–10 month old (mo) and three 17–19 mo male, Dunkin-Hartley retired breeder Guinea pigs were injected via the heart with a single bolus containing 10 kDa rhodamine green tagged and 70 kDa Texas red tagged dextrans (Life Technologies, Carlsbad), prepared and delivered on a per gram basis, *per* previous protocols^14,16,18^ albeit mixed in a single solution prior to injection. Two additional animals (one from each age cohort) served as baseline controls, where one was injected with a single tracer bolus of rhodamine green alone and one was injected with lactated Ringer’s solution (pH 7.3–7.4). Animals were euthanized 5 min after bolus injection.

### Specimen Preparation, Imaging and Analysis

Immediately after euthanasia, knees were resected and frozen (liquid nitrogen) in OCT embedding compound (VWR, Sacramento) before transfer to BioInVision (Cleveland, USA) for concomitant cryosectioning and imaging using an automated microtome-blockface episcopic imaging system (CryoViz™, BioInvision, Cleveland, USA) that allows for microscopic, three dimensional resolution of fluorophores in macroscopic specimens^32^. Serial sections of each sample block (*n* = 3 for each group, 8–10 mo and 17–19 mo old Guinea pigs, respectively) were reconstructed as animated gif files (included as Supplementary Data in the web version of this manuscript, Supplementary Animation 1–3).

## Electronic supplementary material

Dataset 1

Dataset 2

Dataset 3
