# Supplementary figures and images for: Knee Joint Tissues Effectively Separate Mixed Sized Molecules Delivered in a Single Bolus to the Heart
Source: Sci Rep. 2018 Jul 6;8:10254. doi: 10.1038/s41598-018-28228-w (PMC6035244; doi:10.1038/s41598-018-28228-w)

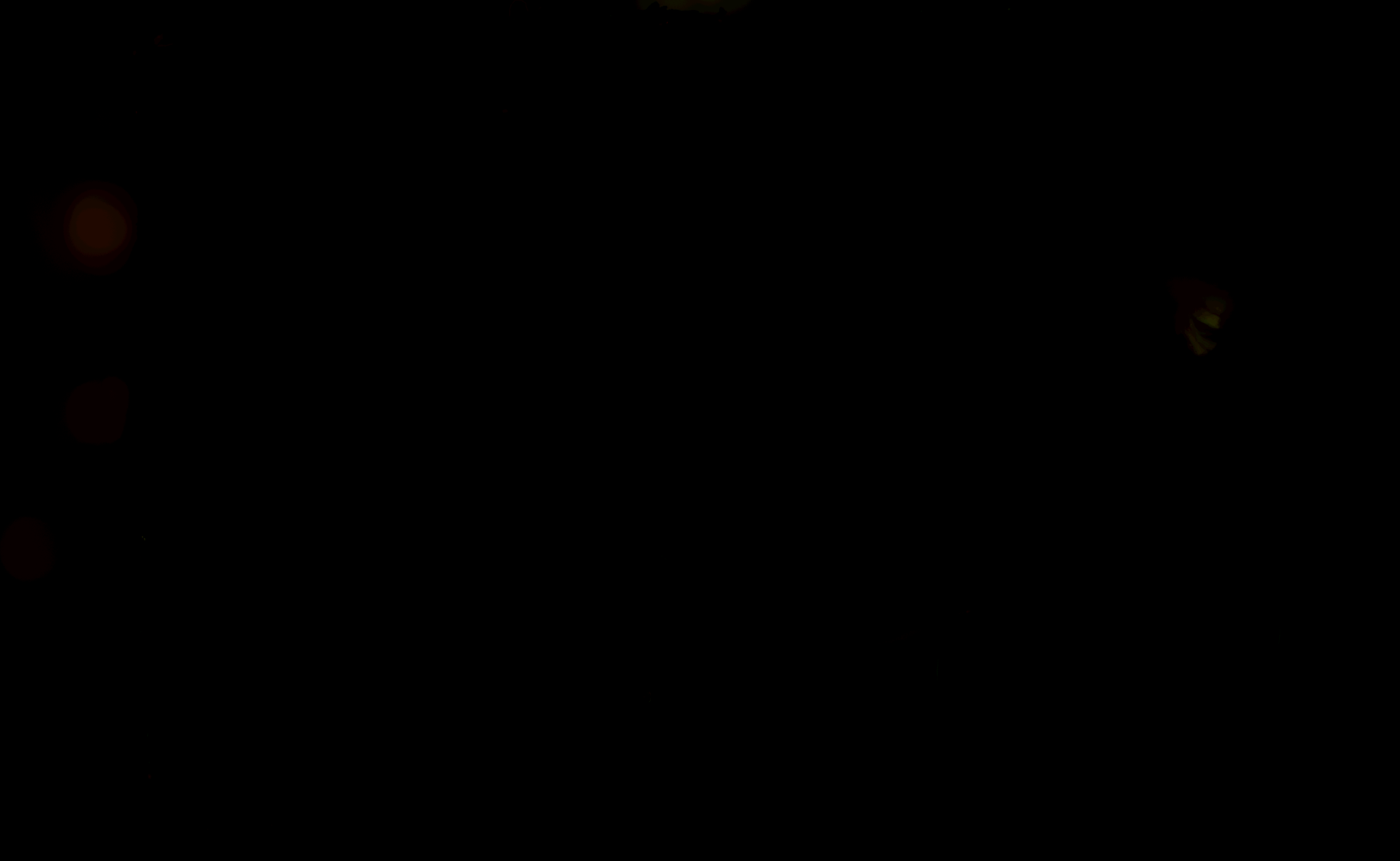

Supplement: Supplementary file 1 — Dataset 1 [file 41598_2018_28228_MOESM1_ESM.zip › Supplementary Figure 1.gif]
